# The prognosis impact of hyperthermic intraperitoneal chemotherapy (HIPEC) plus cytoreductive surgery (CRS) in advanced ovarian cancer: the meta-analysis

**DOI:** 10.1186/s13048-019-0509-1

**Published:** 2019-04-17

**Authors:** Guyu Zhang, Yimin Zhu, Chongdong Liu, Guangming Chao, Ran Cui, Zhenyu Zhang

**Affiliations:** 1 Department of Obstetrics and Gynecology, Beijing Chaoyang Hospital, Capital Medical University, No.8, industrial south road, Chaoyang District, Beijing, China; 2 Department of Oncology, Affiliated Hospital of Guangdong Medical University, Zhanjiang, Guangdong Province China

**Keywords:** HIPEC, Hyperthermic intraperitoneal chemotherapy, CRS, Cytoreductive surgery, Ovarian cancer, Meta-analysis, Review

## Abstract

**Background and objective:**

Previous studies about the prognostic value of the HIPEC have yielded controversial results. Therefore, this study aims to assess the impact of HIPEC on patients with ovarian cancer.

**Results:**

We included 13 comparative studies, and found that the overall survival (OS) and progression-free survival (PFS) in HIPEC groups were superior to groups without HIPEC treatment in the all total population (HR = 0.54,95% CI:0.45 to 0.66, HR = 0.45, 95% CI: 0.32 to 0.62). Additionally, the subgroup analysis showed that patients with advanced primary ovarian cancers also gained improved OS and PFS benefit from HIPEC (HR = 0.59,95% CI:0.46 to 0.75, HR = 0.41,95% CI:0.32 to 0.54). With regard to recurrent ovarian cancer, HIPEC was associated with improved OS (HR = 0.45,95% CI:0.24 to 0.83), but for the PFS, no correlation was observed between HIPC group and the non-HIPEC group (HR = 0.55,95% CI:0.27 to 1.11). HIPEC also led to favorable clinical outcome (HR = 0.64,95% CI:0.50 to 0.82, HR = 0.36,95% CI:0.20 to 0.65) for stage III or IV ovarian cancer with initial diagnosis.

**Conclusion:**

The review indicated that HIPEC-based regimens was correlated with better clinical prognosis for patients with primary ovarian cancers. For recurrent ovarian cancers, HIPEC only improved the OS but did not elicit significant value on the PFS.

**Electronic supplementary material:**

The online version of this article (10.1186/s13048-019-0509-1) contains supplementary material, which is available to authorized users.

## Introduction

Ovarian cancer(OC) is one of the most lethal gynecologic cancers with 22,440 new cases and 14,080 deaths anticipated by 2017 in the United States [1]. Surgery is the optimal treatment for early-stage ovarian cancer, and platinum-based chemotherapy followed by debulking surgery is the standard therapy for advanced ovarian cancer. Although the development of surgery and chemotherapy improved clinical outcomes of patients with advanced ovarian cancer, the 5-year survival rate of less than 30% was still difficult to overcome. Due to the lack of specific clinical symptom and the characteristic of spreading to the abdominal cavity, most of OC have spread to peritoneum by the time of preliminary diagnosis [2]. The natural feature of OC provided a perfect opportunity to develop the local therapy. A systematic review showed that intraperitoneal (IP) chemotherapy prolonged survival time and reduced the risk of death. After every cycle of IP chemotherapy finished, the risk of death decreased by 12% [3]. Despite the positive clinical achievement, a higher rate of adverse events and the frequency of discontinuity hampered the adoption of IP chemotherapy [4].In recent years, Intraperitoneal chemotherapy could be conveyed under hyperthermic circumstances that were termed hyperthermic intraperitoneal chemotherapy (HIPEC). Hyperthermia produced an increased number of lysosomes and lysosomal enzyme activity in malignant cells, resulting in enhanced cancer cell destruction [5]. Moreover, a decreased blood flow or complete vascular stasis were observed in tumors with hyperthermia therapy, which led to accelerated cancer cell death [6]. In contrast to IP chemotherapy without hyperthermia condition, HIPEC had following advantages 1) direct impairment against cancer cells 2) enhancement of the cytotoxicity of chemotherapy 3) inhibition of angiogenesis 4) improvement in denaturation of proteins 5) great tolerance without additional adverse effect [7–11] .However, due to the controversial impact of HIPEC for ovarian cancers, the role of HIPEC in the treatment of ovarian cancer is still debated. In 2015, a published meta-analysis suggested that the CRS + HIPEC +chemotherapy significantly improved 5-year overall survival rate compared to CRS + chemotherapy alone for the patients with primary ovarian cancer, but not for recurrent ovarian cancer [12]. Moreover, the meta-analysis did not provide enough available data to assess the influence of HIPEC on PFS, making it difficult to estimate the clinical benefit of HIPEC comprehensively. On the basis of additional articles, we analyzed all the qualified publications by meta-analysis to evaluate the prognostic impact of HIPEC on patients with ovarian cancers with the goal of identifying the patient population who would be most likely to benefit from HIPEC.

## Methods

### Inclusion and exclusion criteria

In this meta-analysis, comparative clinical trials were included, and the language was restricted to English. Articles were accepted if they complied with the following inclusion criteria: (1) Patients with a diagnosis of advanced primary or recurrent ovarian cancer. (2) Interventions were performed as follows: the experimental group included ovarian cancer patients who were administered by therapy with additional hyperthermic intraperitoneal chemotherapy (HIPEC), and the patients treated with traditional treatment without HIPEC were considered as the control group. (3) The study provides available data to calculate the HR of OS or PFS. Exclusion criteria included (1) Literature reviews, Systematic reviews. (2) Case reports or Case series. (3) Animal Experiments or Cell Experiments. (4) Phase I clinical trial. (5) Duplicate publication. (6) Studies include only the HIPEC group for ovarian cancer.

### Search strategy

Two reviewers independently and simultaneously screened articles in the following databases: PubMed, Embase, Cochrane Library, Clinicaltrials.gov. MeSH terms and entry terms were used to search relevant articles.

The following is an example of the search strategy used on PubMed: (((randomized controlled trial [pt] OR controlled clinical trial [pt] OR randomized [tiab] OR placebo [tiab] OR clinical trials as topic [mesh: noexp] OR randomly [tiab] OR trial [ti]) NOT (animals [mh] NOT humans [mh]))) AND (((((((“Hyperthermia, Induced”[Mesh]) OR ((((((((((((Therapy, Fever) OR Fever Therapy) OR Hyperthermia, Therapeutic)OR Therapeutic Hyperthermia) OR Thermotherapy) OR Induced Hyperthermia) OR Hyperthermic Intraperitoneal Chemotherapy) OR Chemotherapy, Hyperthermic Intraperitoneal) OR Hyperthermic Intraperitoneal Chemotherapies) OR intraperitoneal Chemotherapy, Hyperthermic) OR Hyperthermia, Local) OR Local Hyperthermia))) AND ((“Ovarian Neoplasms”[Mesh]) OR (((((((((((((((((Neoplasm, Ovarian) OR Ovarian Neoplasm) OR Ovary Neoplasms) OR Neoplasm, Ovary) OR Ovary Neoplasm) OR Neoplasms, Ovary) OR Neoplasms, Ovarian) OR Ovary Cancer) OR Cancer, Ovary) OR Cancers, Ovary) OR Ovary Cancers) OR Ovarian Cancer) OR Cancer, Ovarian) OR Cancers, Ovarian) OR Ovarian Cancers) OR Cancer of Ovary) OR Cancer of the Ovary)))) AND surgery)). Reviews of relevant studies were searched manually to find additional eligible studies. All the Publications in these databases are up to May 15, 2018.

### Quality assessment

We estimated studies independently after reading the full text of each study. We used the Cochrane collaboration ROB tool to evaluate the quality of the involved randomized controlled trials (RCTs) [13] (Additional file 1:Table S1). The Newcastle–Ottawa scale (NOS) was employed to assess the quality of the observational study [14] (Additional file 1 :Table S2 ).

### Data extraction

The necessary information of each trial contains the first author’s name, year of publication, country, experimental design, the stage of cancer, mean age, the rate of OS, and the score of completeness of cytoreduction (CC). PFS was defined as the length of time from the start of treatment to the progression of the disease, and OS was defined as the length of time from the beginning of treatment to death. The score of CC was evaluated according to Sugarbaker [15]: CC0: no residual disease; CC1: the residual disease with nodules measuring less than 2.5 mm; CC2: the residual disease with nodules measuring between 2.5 mm and 2.5 cm; and CC3: the residual nodules greater than 2.5 cm. Advanced primary ovarian cancer is defined as the stage of IC-IV in the initial diagnosis.

The hazard ratio (HR) was applied to evaluate the survival effects on PFS and OS. We extracted directly or calculated the hazard ratios and 95% confidence intervals (95% CI) from the survival curve based on the methodology of Tierney [16].

### Statistical analysis

We evaluate all data using Review Manager 5.3 (Cochrane Collaboration) and STATA 12.0 software (Stata Corp., College Station, TX, USA). Inter-study heterogeneity was assessed using the Chi-square test and *I* ^*2*^. A *p*-value > 0.1 or an *I* ^*2*^ < 50% indicate that the heterogeneity is not statistically significant, thus the fixed-effect model is used. When the opposite happens, the random-effect model was applied correspondingly. Furthermore, we make use of sensitivity analysis to analyze the stability of pooled data and exclude studies at high risk of heterogeneity. Publication bias of studies was further appraised by Egger’s tests and Begg’s test.

## Results

### Characteristics of included studies and quality assessment

**Fig. 1:**
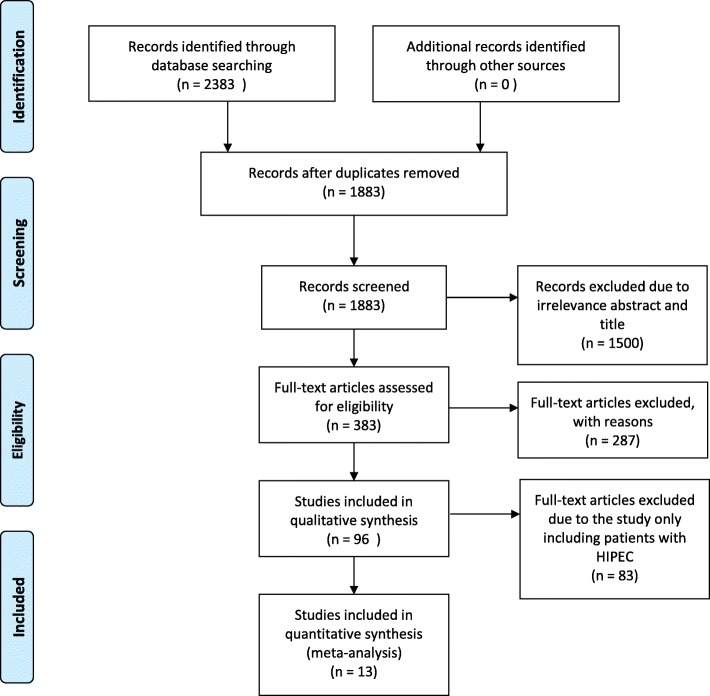
Flow diagram of the literature search strategy

According to the search strategy, we identified a total of 2383 papers. Of these,2370 references including duplication, diagnostic tests, case reports, review, and other irrelevant studies were excluded according to the exclusion criteria. A total of 13 articles were eligible for the meta-analysis. Among the included studies, there were two RCT [17, 18], and eleven observational studies. Our search steps are illustrated in Fig. 1.The general characteristic and scheme of thirteen articles were listed in Table 1 and Additional file 1 :TableS3. The detail of quality assessment for studies is shown in supplementary of Additional file 1 :TableS1 and Additional file 1: Table S2.

**Table 1:** The characteristic of studies included in meta-analysis

Name	Arm	Original	Country	Mean age(year)	Patients (Numbers)	Stage		OS (rate)
						I-II	III-IV	
Ki Sung Ryu2004	Control group	Primary	South Korea	47.7	60	21	39	52.8%(5-year)
	Experimental group			46.1	57	22	35	63.4%(5-year)
J. GORI 2005	Control group	Primary	Argentina	57.0	19	0	19	42.1%(5-year)
	Experimental group			55.5	29	0	29	55.2%(5-year)
FranciscOC2009	Control group	Recurrence	Bulgaria	54	12	0	12	17% (5-year)
	Experimental group			54	14	0	14	57% (5-year)
JIN HWI KIM 2010	Control group	Primary	South Korea	49	24	5	19	25%(8-year)
	Experimental group			48	19	7	12	84.21%(8-year)
Rene Warschkow 2012	Control Group	Primary or Recurrence	Switzerland	65	90	56	35	38.3%(5-year)
	Experimental group			58.9	21	17	4	72.5%(5-year)
Anna Fagotti 2012	Control Group	Recurrence	Italy	55	37	5	32	37.8%(5-year)
	Experimental group			51	30	4	26	76.7%(5-year)
TAMAR SAFRA 2014	Control Group	Recurrence	Israel	54.3	84	7	76	45%(5-year)
	Experimental group			54.3	27	2	25	79%(5-year)
Jean-Franc ¸ois Le Brun 2014	Control Group	Recurrence	France	NR	19	1	18	19.4%(4-year)
	Experimental group			NR	23	2	21	75.6%(4-year)
Cascales-Campos, P. A2014	Control Group	Primary	Spain	57	35	0	35	NR
	Experimental group			57	52	0	52	NR
J. Spiliotis 2015	Control Group	Recurrence	Greece	58.1	60	0	60	18%(3-year)
	Experimental group			58.3	60	0	60	75%(3-year)
Glauco Baiocchi 2016	Control Group	Recurrence	Brazil	58.4	50	10	40	49.5%(5-year)
	Experimental group			51.6	29	2	27	49.7%(5-year)
Alberto A. Mendivil 2017	Control Group	Primary	USA	62.9	69	0	69	75.3%(3-year)
	Experimental group			59.8	69	0	69	82.6%(3-year)
W.J. van Driel 2018	Control Group	Primary	Netherlands	63	122	0	122	38%(5-year)
	Experimental group			61	118	0	118	50%(5-year)

### The association between HIPEC and OS

**Fig. 2:**
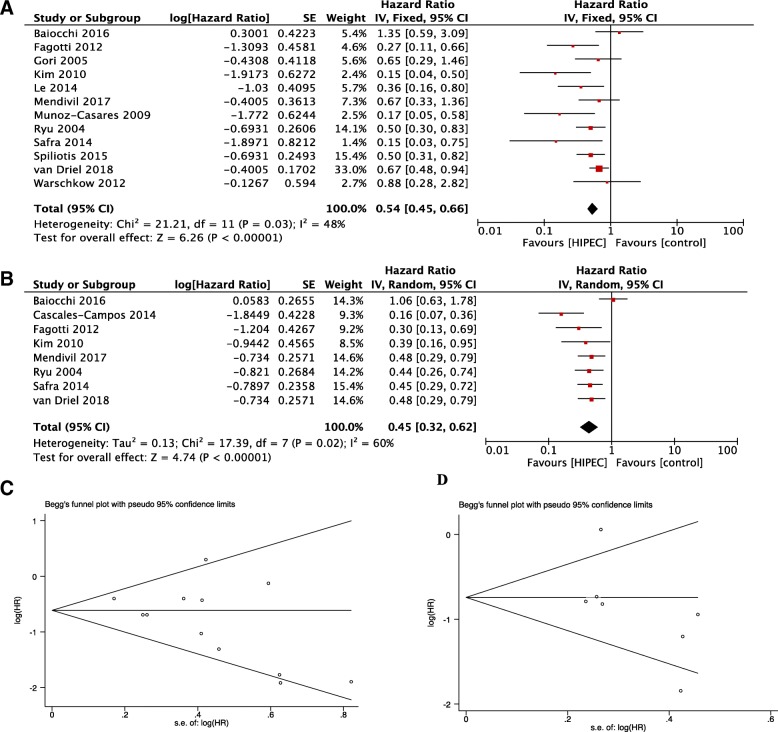
Forest plot assessing prognostic value and funnel plot of Begg’s test between hyperthermic intraperitoneal chemotherapy group and hyperthermic intraperitoneal chemotherapy and **a** forest plot of OS **b** forest plot of PFS **c** funnel plot of OS **d** funnel plot of PFS

Twelve studies were eligible to assess the impact of HIPEC on OS. Pooled data demonstrated that there was an improvement in HIPEC groups compared with the groups without HIPEC treatment in all population (HR = 0.54,95% CI:0.45 to 0.66, *I*^*2*^ = 48%) (Fig.2a). The subgroup analysis indicated that both advanced primary and recurrent patients with ovarian cancers gained significant OS benefit from HIPEC (HR = 0.59,95% CI:0.46 to 0.72, HR = 0.45,95% CI:0.24 to 0.83) (Table 2).

**Table 2:** Summary of the pooled HR

	Studies (*N*)	HR (95%CI)				*I* ^*2*^				*P* value			
		OS	PFS	OS without CC3	PFS without CC3	OS	PFS	OS withoutCC3	PFS withoutCC3	OS	PFS	OS withoutCC3	PFS withoutCC3
HIPEC group vs non-HIPEC group	13	0.54 (0.45-0.66)	0.45(0.32-0.62)	0.51(0.42-0.63)	0.41(0.32-0.52)	48%	60%	39%	10%	<0.0001	<0.0001	<0.0001	<0.0001
Subgroup													
Primary ovarian cancer	6	0.59(0.46-0.75)	0.41(0.32-0.54)	_	_	34%	32%	_	_	<0.0001	<0.0001	_	_
Recurrent ovarian cancer	5	0.45(0.24 to 0.83)	0.55(0.27 to 1.11)	0.38(0.25 to 0.56)	0.41(0.27 to 0.62)	60%	77%	0	0	0.01	0.09	<0.0001	<0.0001
Stage III-IV	5	0.64(0.50 to 0.82)	0.36(0.20 to 0.65)	_	_	0	66%	-	-	0.0004	0.0007	-	-
Interval CRS plus HIPEC	2	0.61(0.45 to 0.83)	0.29(0.1 to 0.86)	-	-	50%	80%	-	-	0.002	0.03	-	-
Primary CRS plus HIPEC	10	0.47(0.37 to 0.61)	0.52(0.41 to 0.65)	0.43(0.33 to 0.55)	0.43(0.33 to 0.55)	50%	50%	28%	0	<0.0001	<0.0001	<0.0001	<0.0001

### The association between HIPEC and PFS

Eight studies provided available data to calculate the HR of the PFS. As shown in Fig. 2b, the pooled data indicated that HIPEC improved PFS significantly compared with patients without HIPEC therapy in all population (HR = 0.45, 95% CI: 0.32 to 0.62). Among primary advanced ovarian cancers, the PFS in the HIPEC group was significantly longer (HR = 0.41, 95% CI:0.32 to 0.54). Interestingly, with regard to recurrent ovarian cancers, although the HIPEC improved the OS significantly, there was no association between HIPEC and PFS (HR = 0.55, 95% CI:0.27 to 1.11).

### The influence of HIPEC in stage III or IV ovarian cancer

Based on the tumor stage, our pooled data also suggested that the prognostic benefit of HIPEC was also observed among patients with stage III or IV(HR = 0.64,95% CI:0.50 to 0.82, HR = 0.36,95% CI:0.20 to 0.65) (Table 2).

### The influence of CC3 in the meta-analysis

In the retrieval process, we found that only studies of Warschkow and Baiocchi [19, 20] were reported to include patients with CC3, however, Warschkow eliminated patients with CC3 when they calculated the HR. When we excluded the Baiocchi’s study, the *I*^*2*^ of the pooled data decreased obviously, even the HIPEC showed useful effect on PFS of recurrent ovarian cancer, which was opposite to our pooled data (Table 2). The controversy result showed that the CC3 contributed to the high heterogeneity of pooled data and displayed a critical role in the therapy value of HIPEC.

### The influence of the CRS plus HIPEC timing in the meta-analysis

As illustrated in Table 2, both primary HIPEC plus CRS followed by chemotherapies and interval combination of HIPEC and CRS after adjuvant chemotherapies indicated improved prognostic effect on OS (HR = 0.61, 95% CI:0.45 to 0.83, HR = 0.47, 95% CI:0.37 to 0.61) and PFS (HR = 0.29, 95% CI:0.1 to 0.86, HR = 0.52, 95% CI:0.41 to 0.65).

### Sensitive analysis

**Fig. 3:**
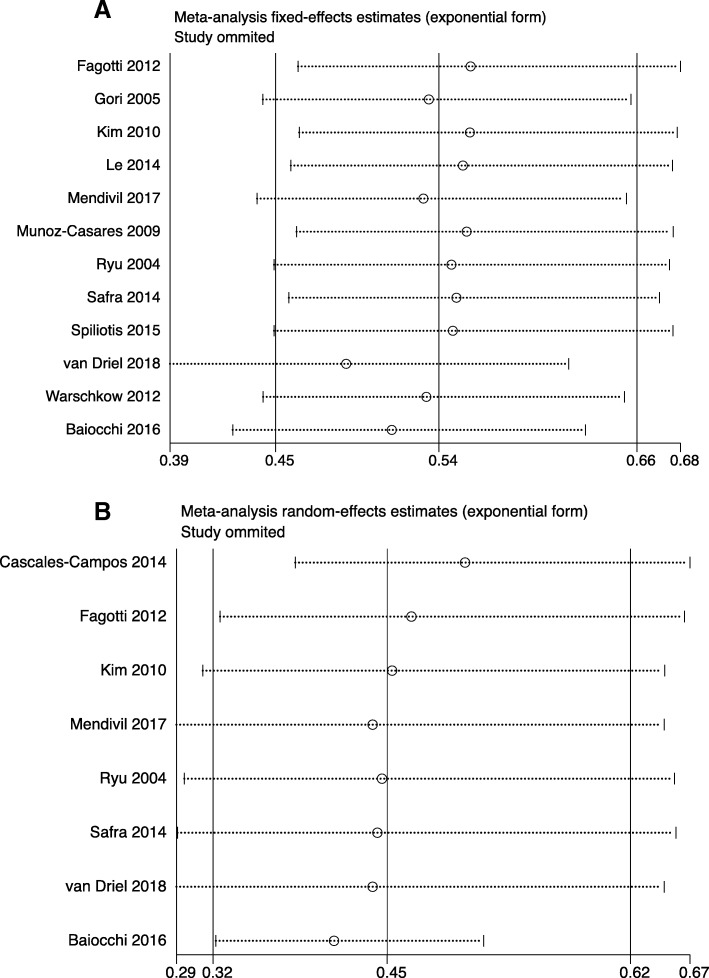
Sensitive analysis between HIPEC group and non-HIPEC group **a** OS **b** PFS

To investigate the impact of the individual study on the pooled data, we conducted a sensitivity analysis in which every study was deleted consecutively to test the stability of the data. The result of OS and PFS was robust, sequential omission of data from any individual study did not affect the results (Fig. 3a-b).

### Publication bias

As shown in Fig. 2c-d, the Begg’s test and Egger’s test were applied to evaluate the bias of publication, and there was no significant bias in PFS (*P*B = 0.216, *P*E = 0.147) as well as OS (*P*B = 0.086, *P*E = 0.097).

## Discussion

To date, there is increasing evidence that the combination of CRS and HIPEC enhances the prognosis of ovarian cancer significantly [19, 21–27]. However, in recent years, some studies demonstrated that the HIPEC did not show any improvement in OS compared with the therapy without the HIPEC [20, 28]. Thus, whether HIPEC offered benefit to patients is still under debate. Although the previous meta-analysis had revealed the association between HIPEC and better clinical prognosis, the positive effect was only applicable to the primary advanced ovarian cancer. This time, we made use of HR [29] instead of OR in the previous meta-analysis to describe the prognostic effect of HIPEC. Moreover, we found that a total of eight studies have qualified data to calculate the HR of PFS which omitted in the previous meta-analysis. The current meta-analysis demonstrated that the HIPEC not only improved OS significantly but also prolonged the PFS in all population. Subgroup analysis indicated that HIPEC was associated with better clinical outcome whether primary or recurrent patients. Even stage III or IV ovarian cancer patients could benefit from HIPEC. Noticeably, deleting the study including patients with CC3 could result in the decrease of heterogeneity (Table 2), which was consistent to the previous literatures that the score of CC was one of the most critical prognostic factors in advanced ovarian cancer when HIPEC followed a cytoreductive surgery [30, 31].

There were some limitations in the current meta-analysis. First, we searched the publications as complete as possible, only papers published in English were eligible, which may lead to selection bias. Secondly, the shortage of RCT was likely to increase the risk of bias. Thirdly, most of the studies were from observational studies, which might compromise the meta-analysis. Fourthly, factors including whether ovarian cancer resists platinum or not, pathological classification of ovarian cancer, the agency of chemotherapy medicine, and the completeness of cytoreduction were needed to be stratified further to determinate the most suitable candidates for HIPEC. Additionally, among 13 included studies, only van Driel reported the information about adverse events (AE) between HIPEC arm and non-HIPEC arm. There were no significant differences of AE between the two groups. For the HIPEC treatment group, the most common AE included Abdominal pain, Nausea, Vomiting, Fatigue, Pain. Most common AE of grade 3 or 4 were Abdominal pain, infection, ileus [18]. Kim [24] and Mendivil [28] also reported a similar situation of toxicity and AE on HIPEC therapy. Finally, we hope that more RCT and well-designed observational studies are incorporated into the meta-analysis to ascertain and evaluated the effect and the toxicity of HIPEC in ovarian cancer.

## Conclusion

Summary, HIPEC-based regimens might result in favorable PFS and OS for patients with advanced primary ovarian cancer. With regards to recurrent ovarian cancers, HIPEC only improved the OS but did not elicit positive value on the PFS. Additionally, it was associated with better clinical prognostic outcome among Stage III or IV ovarian cancer patients with the initial diagnosis. The CC3 might display a critical role in reducing the effect of HIPEC.

## Additional file

Additional file 1: **Table S1**. Quality assessment of included RCT. **Table S2**.The Newcastle-Ottawa scale(NOS)scores of the included non-RCTs. **Table S3.** The scheme of studies included in meta-analysis. (DOCX 24 kb)

## Abbreviations

CC: Completeness of cytoreduction
CI: Confidence interval
CRS: Cytoreductive surgery
HIPEC: The adenomatous polyposis coli
HR: Hazard ratio
OS: Overall survival
PFS: Progression free survival
RCT: Randomized controlled trial
