# Supplementary material for: The prognosis impact of hyperthermic intraperitoneal chemotherapy (HIPEC) plus cytoreductive surgery (CRS) in advanced ovarian cancer: the meta-analysis
Source: J Ovarian Res. 2019 Apr 17;12:33. doi: 10.1186/s13048-019-0509-1 (PMC6472063; doi:10.1186/s13048-019-0509-1)
Supplement: Supplementary file 1 — Table S1. Quality assessment of included RCT. Table S2.The Newcastle-Ottawa scale(NOS)scores of the included non-RCTs. Table S3. The scheme of studies included in meta-analysis. (DOCX 24 kb) [file 13048_2019_509_MOESM1_ESM.docx]

Table S1 Quality assessment of included RCT

| Citation | Random sequence generation | Allocation concealment | Blinding of participants and personnel | Blinding of outcome assessment | Incomplete outcome data | Selective reporting | Other bias |
| --- | --- | --- | --- | --- | --- | --- | --- |
| W.J.vanDriel 2018 | Low risk | Low risk | High risk | Unknown risk | Low risk | Low risk | Unknown risk |
| J. Spiliotis 2015 | Low risk | Low risk | Low risk | Unknown risk | Unknown risk | Low risk | Unknown risk |

TableS2 The Newcastle-Ottawa scale(NOS)scores of the included non-RCTs

| Cohort study | Representatives of the exposed cohort | Selection of the non-exposed cohort | Ascertainment of exposure | Demonstration that outcome of interest was present at start of study | Comparability of cohorts on the basis of the design or analysis | Assessment of outcome | Was follow-up long enough for outcomes to occur | Adequacy of follow up of cohorts | Total |
| --- | --- | --- | --- | --- | --- | --- | --- | --- | --- |
| Ki Sung Ryu 2004 | 1 | 1 | 1 | 1 | 1 | 1 | 1 | 1 | 8 |
| J. GORI 2005 | 1 | 1 | 1 | 1 | 0 | 1 | 1 | 1 | 7 |
| Francisco C 2009 | 1 | 1 | 1 | 1 | 0 | 1 | 1 | 0 | 6 |
| JIN HWI KIM 2010 | 1 | 1 | 1 | 1 | 1 | 1 | 1 | 1 | 8 |
| Rene Warschkow 2012 | 1 | 1 | 1 | 1 | 1 | 1 | 1 | 0 | 7 |
| Anna Fagotti 2012 | 1 | 1 | 1 | 1 | 0 | 1 | 1 | 0 | 6 |
| TAMAR SAFRA 2014 | 1 | 1 | 1 | 1 | 1 | 1 | 1 | 0 | 7 |
| Jean-Franc ̧ois Le Brun 2014 | 1 | 1 | 1 | 1 | 1 | 1 | 1 | 0 | 7 |
| Cascales-Campos, P. A.2014 | 1 | 1 | 1 | 1 | 1 | 1 | 1 | 0 | 7 |
| Glauco Baiocchi2016 | 1 | 1 | 1 | 1 | 0 | 1 | 1 | 0 | 6 |
| Alberto A. Mendivil 2017 | 1 | 1 | 1 | 1 | 1 | 0 | 1 | 1 | 7 |

**Table S3 The scheme of studies included in meta-analysis**

|  |  |  |  |  |  |  |
| --- | --- | --- | --- | --- | --- | --- |
| Name | arm | Scheme | HIPEC agent | chemotherapy agent | The completeness of cytoreduction | |
|  |  |  |  |  | CC0-CC2 | CC3 |
| Ki Sung Ryu2004 | Control  group | Cytoreductive surgery+ chemotherapy | Carboplatin  Or interferon-a | Platinum-based chemotherapy | NR | NR |
|  | Experimental group | Cytoreductive Surgery + chemotherapy  +HIPEC |  | Platinum-based chemotherapy | NR | NR |
| J. GORI 2005 | Control  group | Cytoreductive surgery +chemotherapy | Cisplatin | Cisplatin and cyclophosphamide | 19 | 0 |
|  | Experimental group | Cytoreductive Surgery + chemotherapy+ HIPEC |  | Cisplatin and cyclophosphamide | 29 | 0 |
| FranciscOC2009 | Control  group | Cytoreductive Surgery+ chemotherapy | Paclitaxel | NR | 12 | 0 |
|  | Experimental group | Cytoreductive Surgery +HIPEC+ chemotherapy |  | cisplatin or carboplatin and paclitaxel | 14 | 0 |
| JIN HWI KIM 2010 | Control  group | Cytoreductive Surgery + chemotherapy | Paclitaxel | Platinum and taxanes or Platinum and cyclophosphamide | 24 | 0 |
|  | Experimental group | Cytoreductive Surgery + chemotherapy+ HIPEC |  | Platinum and taxane or Platinum and cyclophosphamide | 19 | 0 |
| Rene Warschkow 2012 | Control  Group | Cytoreductive Surgery+ chemotherapy | Cisplatin | Carboplatin or Taxol or Endoxan | 90 | 21 |
|  | Experimental group | Cytoreductive Surgery + HIPEC+ chemotherapy |  | Carboplatin or Taxol or Endoxan |  |  |
| Anna Fagotti 2012 | Control  Group | Cytoreductive Surgery+ chemotherapy or exclusive chemotherapy | Oxaliplatin | NR | 37 | 0 |
|  | Experimental group | Cytoreductive Surgery + HIPEC+ chemotherapy |  | NR | 30 | 0 |
| TAMAR SAFRA 2014 | Control  Group | chemotherapy | Cisplatin and doxorubicin or paclitaxel and carboplatin or cisplatin and mitomycin‐C | carboplatin and paclitaxel, pegylated liposomal doxorubicin, gemcitabine, or topotecan | 84 | 0 |
|  | Experimental group | surgical reduction + HIPEC |  | NR | 27 | 0 |
| Jean-Franc ̧ois Le Brun 2014 | Control  Group | Chemotherapy +surgical reduction | cisplatin or eloxatin or mitomycin | Platinum-based chemotherapy | 23 | 0 |
|  | Experimental group | Chemotherapy +surgical reduction + HIPEC |  | Platinum-based chemotherapy | 19 | 0 |
| Cascales-Campos, P. A2014 | Control Group | Chemotherapy +surgical reduction | paclitaxel | platinum and taxanes | 35 | 0 |
|  | Experimental group | Chemotherapy +surgical reduction + HIPEC |  | platinum and taxanes | 52 | 0 |
| J. Spiliotis 2015 | Control  Group | Surgical reduction + chemotherapy | Cisplatin and paclitaxel or Doxorubicin and (paclitaxel or mitomycin) | NR | 60 | 0 |
|  | Experimental group | Surgical reduction +HIPEC+ chemotherapy |  | NR | 60 | 0 |
| Glauco Baiocchi 2016 | Control  Group | surgical reduction | Mitomycin C and cisplatin or cisplatin (50 mg/m2) and doxorubicin or cisplatin alone or oxaliplatin alone | NR | 44 | 6 |
|  | Experimental group | Surgical reduction +HIPEC |  | NR | 28 | 1 |
| Alberto A. Mendivil 2017 | Control  Group | surgical reduction +chemotherapy + HIPEC | carboplatin | paclitaxel and carboplatin | 69 | 0 |
|  | Experimental group | surgical reduction +chemotherapy |  | paclitaxel and carboplatin | 69 | 0 |
| W.J. van Driel  2018 | Control  Group | chemotherapy +surgical reduction+ chemotherapy | cisplatin | carboplatin and paclitaxel | 122 | 0 |
|  | Experimental group | chemotherapy +surgical reduction + HIPEC+ chemotherapt |  | carboplatin and paclitaxel | 118 | 0 |

**NR:** no report
